# The Effect of Depth of Anesthesia on Postoperative Pain in Laparoscopic Sleeve Gastrectomy: A Randomized Controlled Trial

**DOI:** 10.1007/s11695-024-07207-3

**Published:** 2024-04-08

**Authors:** Xue Zhang, Xin-Yue Chen, Rui-Jia Gao, Yu Huang, Shi-Meng Mao, Ji-Ying Feng

**Affiliations:** Department of Anesthesiology, The Affiliated Lianyungang Hospital of Xuzhou Medical University, No. 6 Zhenhua East Road, Lianyungang, 222002 Jiangsu China

**Keywords:** Anaesthetic depth, Acute postoperative pain, Obesity, Laparoscopic sleeve gastrectomy

## Abstract

**Background:**

Patients with obesity are more sensitive to pain and more likely to have acute postoperative pain (APP). Studies have shown that the depth of anesthesia may affect the incidence of APP. The purpose of the study was to look into the connection between APP and depth of anesthesia in patients with obesity undergoing laparoscopic sleeve gastrectomy.

**Methods:**

This is a prospective, double-blinded randomized clinical trial, 90 patients undergoing laparoscopic sleeve gastrectomy were randomly divided into two groups: the light anesthesia group (Bispectral Index of 50, BIS 50) and the deep anesthesia group (BIS 35). The degree of pain was evaluated by the visual analogue scale (VAS) at 0, 12, 24, 48, and 72 h after surgery. The use of analgesics, grade of postoperative nausea and vomiting (PONV), and the Quality of Recovery-15 (QoR-15) score were recorded.

**Results:**

The VAS scores at rest or coughing at 0, 12, and 24 h after surgery in the BIS 35 group were lower than those in the BIS 50 group (*P* < 0.05). Fewer patients in the deep anesthesia group needed analgesia during the recovery period, and patient satisfaction was higher on the 3rd day after surgery (*P* < 0.015, *P* < 0.032, respectively).

**Conclusions:**

For patients with obesity, maintaining a deeper depth of anesthesia during surgery is beneficial to reduce APP causes less need for additional analgesic drugs, and improves patient satisfaction.

**Graphical Abstract:**

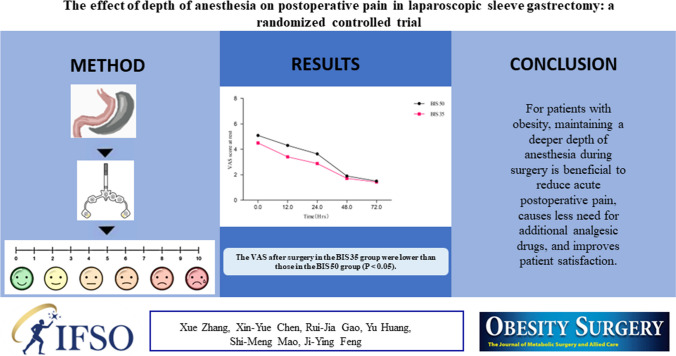

## Introduction

With a changing food source and a more sedentary lifestyle, the prevalence of obesity has increased globally, especially in China [1]. With the increase in the obese population, a growing number of patients with obesity are seen in elective or emergency surgery [2]. Obesity is a high-risk factor for a variety of diseases, including metabolic syndrome, diabetes mellitus, obstructive sleep apnea, gastroesophageal reflux syndrome, and cardiovascular disease. Patients with obesity suffer from higher perioperative risk and have a higher incidence of postoperative complications when undergoing surgery [3, 4]. One large-scale retrospective study found that obesity was an independent cause of postoperative pain requiring care [5]. In a review conducted in 2023 on obesity and pain, it was found that patients who have obesity are more likely to have increased pain sensitivity and altered pain threshold due to certain neuroendocrine mechanisms [6]. This may result in a low-grade inflammatory condition which is linked to the activity of white adipose tissue that is caused by chronic activation of both the innate and adaptive immune systems [7–9]. Therefore, the incidence of acute postoperative pain (APP) in patients with obesity is higher and the degree of pain is more severe [10]. More than 80% of surgical patients experienced acute postoperative pain, and about 75% of patients reported moderate, severe, or extremely severe postoperative pain [11]. APP not only delays the recovery of patients, increases the incidence of pulmonary complications, but also reduces patient satisfaction [12, 13]. How to reduce the occurrence of APP and increase the postoperative comfort of patients is a major focus of anesthesia.

In recent years, with the popularity of anesthesia depth monitoring equipment like the Narcotrend Index and Bispectral Index (BIS), it is now feasible to monitor anesthesia depth and implement personalized anesthesia depth management during general anesthesia, which reduces the consumption of anesthetics and promotes the early recovery after surgery [14, 15]. The BIS value fluctuates from 0 to 100, the smaller the value, the deeper the depth of anesthesia. Meanwhile, anesthesiologists are starting to pay more attention to the connection between the depth of anesthesia and the prognosis following surgery, such as stress reactions, postoperative cognitive dysfunction, mortality, etc. [16–18]. Some studies have shown that maintaining a deeper depth of anesthesia during surgery may reduce APP in patients [12, 19, 20]. However, there are few studies on patients with obesity, so the purpose of this study is to explore the effect of different depths of anesthesia on postoperative pain in patients with obesity.

## Methods and Materials

The trial received approval on December 16, 2022, from the affiliated Lianyungang Hospital of Xuzhou Medical University Ethics Committee (Ethical Application Reference: KY-20221123002-01). On May 11, 2023, the Chinese Clinical Trial Registry has it listed (ChiCTR2300071313). The study's protocol complied with CONSORT recommendations. Written informed consent was signed by every participant.

The inclusion criteria of this study: 18–65 years old, ASA grade I–III, BMI 28–55 kg/m^2^, elective laparoscopic sleeve gastrectomy patients. Patients were excluded if they had known severe cardiac, liver and renal dysfunctions, history of mental illness, alcoholism, drug abuse, and chronic pain, history of gastrointestinal surgery, allergy to drugs used in the surgery, and other conditions that the investigators consider to be inappropriate to participate in this trial. Patients with severe perioperative complications and postoperative follow-up data loss were also excluded from this trial.

Using a randomization process created by a statistician, all of the included patients were divided into two groups at a 1:1 ratio: light anesthesia group (BIS 50) and deep anesthesia group (BIS 35). The grouping information was enclosed in opaque envelopes, which could only be disclosed when performing anesthesia induction. Both patients and follow-up investigators were blinded to the grouping.

Standardized monitoring processes were initiated on arrival in the operation room. Anesthesia was induced with intravenous 1 μg/kg dexmedetomidine, 2–3 mg/kg propofol, 0.15–0.25 mg/kg cisatracurium, and 0.3–0.5 μg/kg sufentanil. The anesthesia depth needed to be changed to the objective value within ten minutes after the skin incision in accordance with the grouping. Pursuing BIS objectives at the price of patient safety was not allowed. Propofol and remifentanil infusion rates were modified during the procedure in accordance with the BIS target established in the sealed envelope.

Total intravenous anesthesia was used during the surgery. In order to prevent vomiting, all patients received dexamethasone 10 mg and palonosetron hydrochloride 0.25 mg intravenously. In addition, in order to reduce the awakening pain, 40 mg parecoxib sodium was given half an hour before the end of the surgery. Each patient received local infiltration anesthesia with 0.75% ropivacaine before the skin closure. Patients were ventilated with pure oxygen to restore spontaneous respiration and the endotracheal tube was extubated in accordance with the indication of extubation.

All patients were equipped with patient-controlled intravenous analgesia (PCIA) pump using 2 µg/kg sufentanil and 0.5 mg palonosetron hydrochloride in 100 mL saline. The analgesia pump's parameters were set to 2 mL per hour as the background infusion. The mean arterial pressure (MAP), heart rate (HR), peripheral oxygen saturation (SpO2), and BIS were recorded at 5-min intervals during the maintenance period. At the same time, the intraoperative drug use, the anesthesia conditions, and operative complications were recorded during the surgery. All the recordings were put back into the sealed envelope after the surgical procedure.

The primary outcome of the trial was the degree of pain, which was evaluated by visual analogue scale (VAS) at 0, 12, 24, 48, and 72 h when returned to the ward. Secondary outcomes include the extra use of analgesics, patient satisfaction, Quality of Recovery-15 (QoR-15) score, and postoperative nausea and vomiting (PONV). In the initial three days after surgery, the patients were followed up in the ward. If the patient's VAS score is > 4, additional parecoxib sodium 40 mg will be given for analgesia.

A 10-point rating system was used to gauge the pain's intensity, with 0 indicating no pain, and 10 indicating the maximal pain. The PONV grade was used to record and evaluate postoperative nausea and vomiting, with grade I indicating no nausea and vomiting, and IV indicating severe nausea (feel nausea and vomiting stomach contents). Overall satisfaction was assessed using a 0–10 scale (where 0 represents extremely dissatisfied and 10 extremely satisfied) within 72 h.

It should be noted that the dose calculation methods of all drugs used in this trial refer to the Guidelines of Association of Anesthetists of Great Britain and Ireland Society for Obesity and Bariatric Anesthesia. Sufentanil, remifentanil, cisatracurium (maintenance dose), propofol (maintenance dose) and intraoperative infusion volume were calculated by lean body weight. Propofol (load dose), cisatracurium (load dose), dexmetomidine, and sufentanil (analgesia pump) were calculated by corrected body weight, and tidal volume was calculated according to ideal body weight.

According to the published data, 72 patients in a t-test with a two-sided alpha of 5% and 90% power were required. Taking into account the 1:1 ratio, we projected a 20% dropout rate. Ultimately, 90 patients were included in the study.

The statistical software SPSS 25.0 (IBM, New York, USA) was used to process the data. Regularly distributed data were shown as means; irregularly distributed data were shown as medians; categorical data were shown as frequencies. Additionally, for categorical data, a risk ratio with a 95% confidence interval was displayed. Independent two-sample t-tests and Pearson's χ^2^ tests were used to compare the normally distributed data and categorical data, respectively. The Mann-Whitney U-test was used for continuous variables with a non-normal distribution. *P* values on both sides of less than 0.05 were considered to be statistically significant.

## Results

**Fig. 1:**
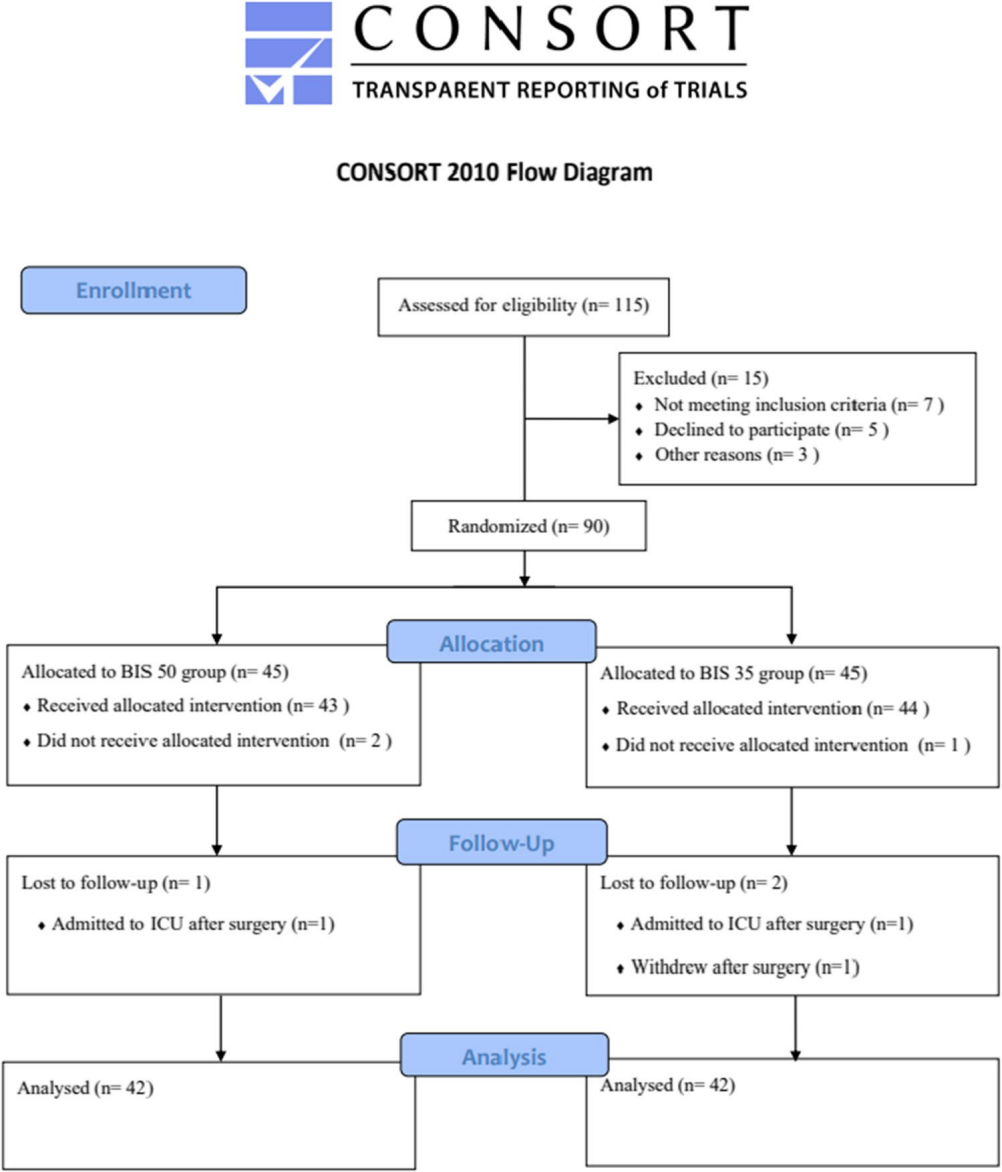
CONSORT flow diagram for the study

Figure 1 shows the participant flow diagram. There were 90 participants in the trial, and they were divided into two groups of light anesthesia group (BIS 50) and deep anesthesia group (BIS 35). The study was not completed by one patient in the BIS 35 group and two patients in the BIS 50 group. In addition, some patients received ICU treatment, and some patients withdrew their informed consent after surgery. Ultimately, 84 patients were included in the analysis.

Baseline patient characteristics are shown in Table 1. The patients' mean age was 31.36 ± 7.67 years. Among them, 28.6% were men and 71.4% were women, and 55% of women had polycystic ovary syndrome. None of the examined baseline variables showed any differences between the groups.

**Table 1:** The preoperative characteristics

	BIS 50 group	BIS 35 group	*P* value
Age (yr)	31.26 ± 8.85	31.45 ± 6.30	0.910
Sex, males	12 (28.57%)	12 (28.67%)	1.000
Height (m)	1.69 ± 0.07	1.68 ± 0.07	0.702
Weight (kg)	109.51 ± 19.70	105.55 ± 19.21	0.934
LBW (kg)	59.47 ± 11.23	58.30 ± 11.36	0.635
ABW (kg)	83.02 ± 12.12	81.08 ± 12.00	0.463
IBW (kg)	65.36 ± 9.06	64.76 ± 8.77	0.760
BMI (kg/m^2^)	38.20 ± 5.02	37.04 ± 4.59	0.271
ASA grade, n (%)			
I	0	1 (2.4%)	0.141
II	28(66.7%)	34(81.0%)	
III	14(33.3%)	7(16.7%)	
State of health, n (%)			
Hypertension	3(7.1%)	4(9.5%)	0.693
Diabetes mellitus	6(14.3%)	4(9.5%)	0.500
Sleep apnea	8(19.0%)	9(21.4%)	0.786
Polycystic ovary syndrome	15(40.5%)	16(42.1%)	0.892
Hyperuricemia	6(14.3%)	5(11.9%)	0.746

*LBW* Lean body weight, *ABW* Adjusted body weight, *IBW* Ideal body weight, *BMI* Body mass index, *ASA* American Society of Anesthesiologists

**Fig. 2:**
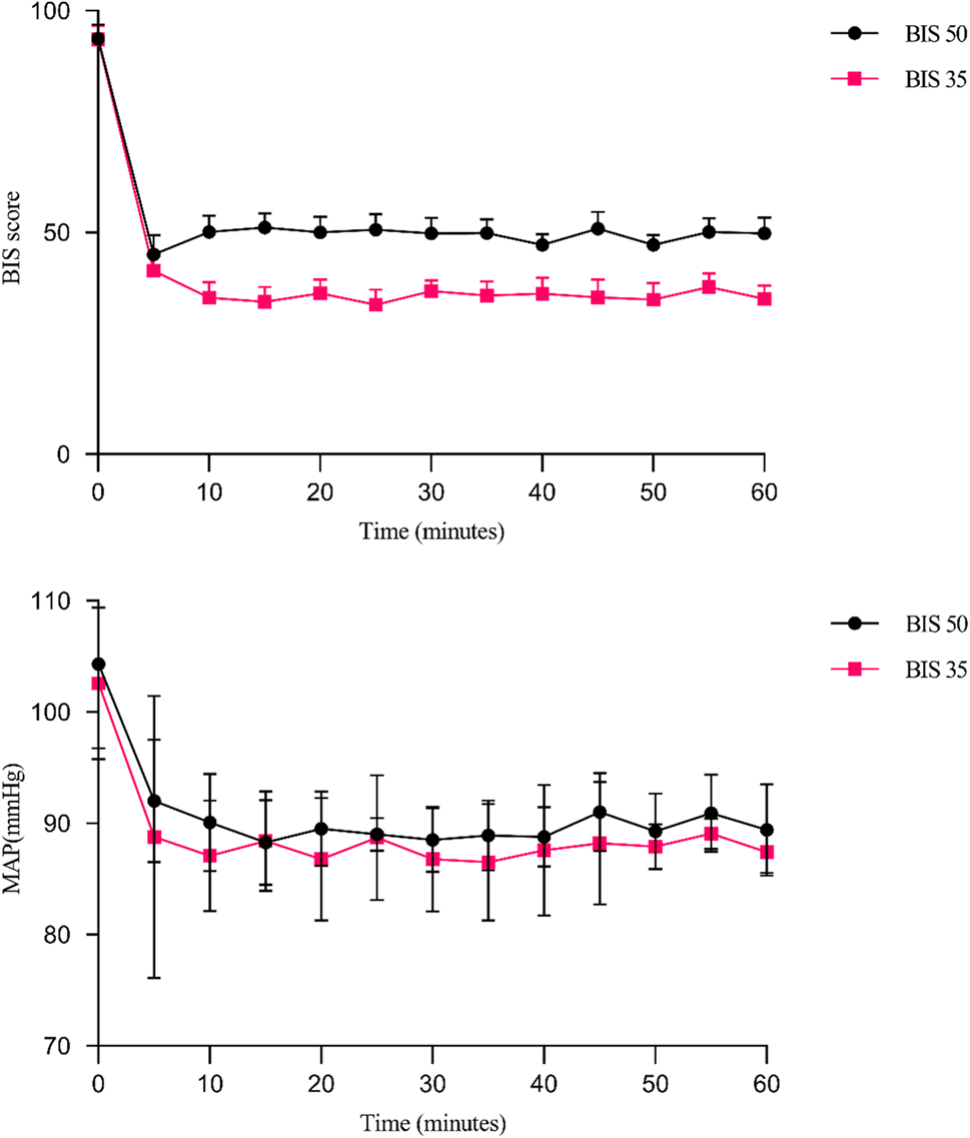
BIS and MAP values in two groups

Intraoperative characteristics are shown in Table 2. BIS and MAP values were compared between the two groups (Fig. 2). The average BIS values for the BIS 50 group and the BIS 35 group were 49.71 ± 4.1 and 35.60 ± 4.8, respectively (*P* < 0.001). In the BIS 35 group, the total propofol dosages were considerably higher (*P* = 0.041). There were no differences between the groups in the length of anesthesia or surgery, infusion volume, the use of nitroglycerin, norepinephrine, or cisatracurium.

**Table 2:** The intraoperative characteristics

	BIS 50 group	BIS 35 group	*P* value
BIS	49.71 ± 4.1	35.60 ± 4.8	< 0.001**
MAP	91.45 ± 9.92	88.12 ± 9.21	0.114
Perioperative medications			
Dexmedetomidine (mg)	83.02 ± 12.12	81.08 ± 12.00	0.463
Sufentanil (μg)	25(20–25)	20(20–25)	0.293
Propofol (mg)	965.30 ± 280.03	1114.73 ± 387.41	0.046*
Cisatracurium	20(18–20)	10(16–20)	0.258
Remifentanil (mg)	0.68 ± 0.21	0.72 ± 0.22	0.384
Use of norepinephrine n (%)	4(9.5%)	8(19.0%)	0.212
Use of nitroglycerin n (%)	1(2.4%)	0	0.314
Time, mean (min)			
Anesthesia	116(103–128)	117(102–135)	0.558
Surgery	83(69–95)	79(68–100)	0.645
Infusion volume (mL)	1000(875–1200)	1000(875–1400)	0.299

*BIS* Bispectral Index, *MAP* Mean arterial pressure

*P < 0.05, **P < 0.001

**Fig. 3:**
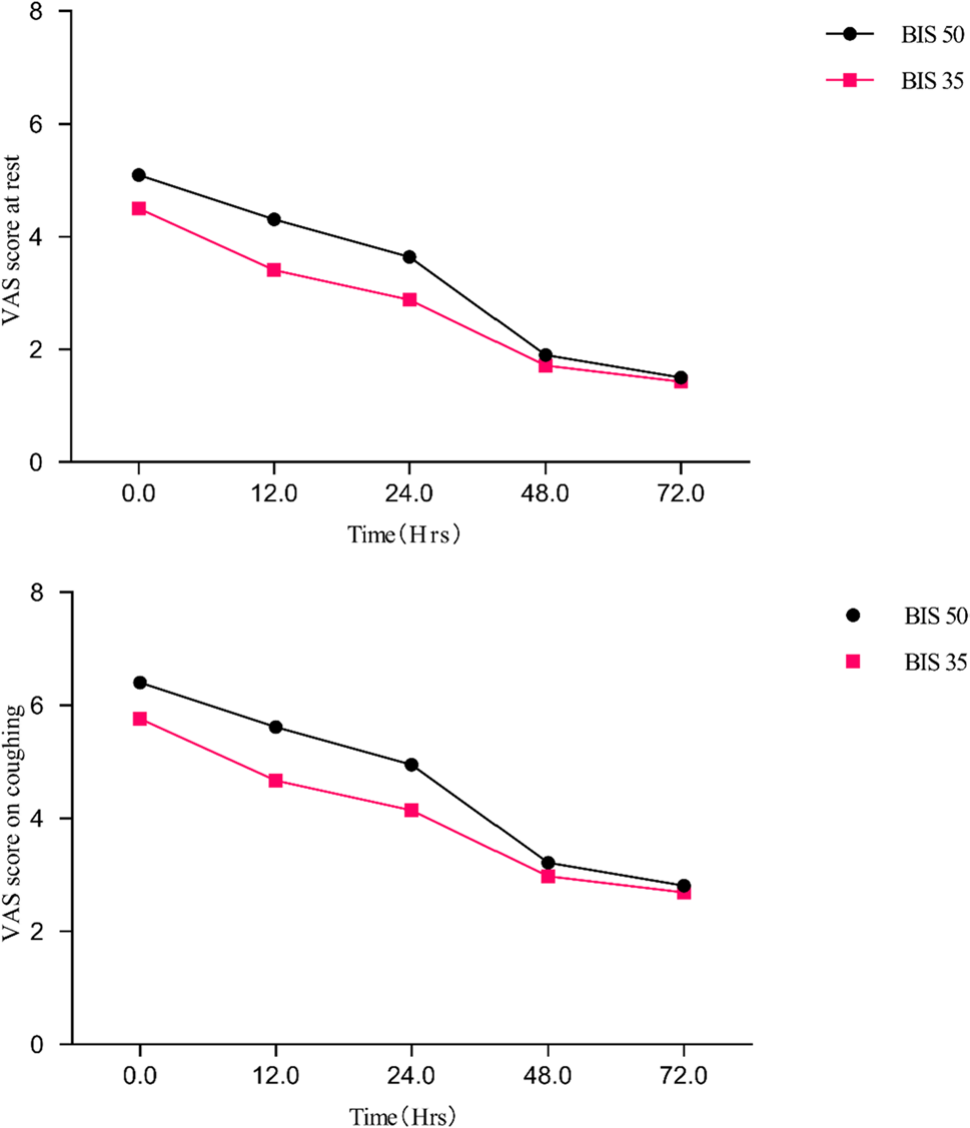
Comparison of VAS score at rest and coughing

Figure 3 shows the comparison of VAS scores at rest and coughing. At any time point, the VAS score in the BIS 35 group was lower than that in the BIS 50 group. At 0 h, the VAS score in the BIS 50 group was 5.10 ± 1.12 at rest, and 6.40 ± 1.43 at coughing. The VAS score in the BIS 35 group was 4.50 ± 1.73 at rest, and 5.76 ± 1.46 at coughing. At 12 h, the mean VAS scores at rest and coughing were 4.31 ± 1.33 and 5.62 ± 1.51, respectively, in the BIS 50 group and 3.40 ± 1.19 and 4.67 ± 1.41, respectively, in the BIS 35 group. At 24 h, the mean VAS scores at rest and coughing were 3.64 ± 1.45 and 4.95 ± 1.61, respectively, in the BIS 50 group and 2.88 ± 1.27 and 4.14 ± 1.47, respectively, in the BIS 35 group. Pain intensities at rest or coughing in the BIS 35 group patients at 0, 12, and 24 h were significantly lower than those in the BIS 50 group (*P* < 0.05). At 48 and 72 h, the VAS score at rest and coughing in the BIS 35 group was lower than that in the BIS 50 group, but the difference was not statistically significant.

Postoperative outcomes are shown in Table 3. In the initial three days after surgery, there are fewer patients in the deep anesthesia group needed additional analgesia, and patient satisfaction was higher at the time of recovery (*P* < 0.015, *P* < 0.032, respectively). In addition, as for the postoperative anesthesia recovery, in the BIS 50 group, the extubation time and PACU stay time were shorter after surgery, and the difference was statistically significant (*P* < 0.001 and *P* = 0.038, respectively). No statistical differences in the postoperative QoR-15 scores and the PONV grade at 24 h after surgery were observed in both groups.

**Table 3:** Postoperative outcomes

	BIS 50 group	BIS 35 group	*P* value
Time to extubation (min)	3.62 ± 3.55	7.26 ± 4.53	< 0.001**
Time in PACU (min)	38.55 ± 6.83	42.36 ± 9.24	0.035*
Overall satisfaction	6.88 ± 1.09	7.38 ± 1.01	0.032*
Rescue analgesia(%)	30(71.4%)	19(45.2)	0.015*
QoR-15 score	0.52 ± 0.92	0.26 ± 0.70	0.145
24 h	127(121–131)	128(120–135)	0.412
48 h	138(132–138)	138(135–138)	0.529
72 h	145(142–145)	145(142–145)	0.577
PONV grade			
I	7(16.7%)	11(26.2%)	0.708
II	15(35.7%)	15(35.7%)	
III	13(31.0%)	11(26.2%)	
IV	7(16.7%)	5(11.9)	

*PACU* Postoperative anesthesia care unit, *QoR-15* Quality of Recovery-15, h, hours, *VAS* Visual analogue scale, *PONV* Postoperative nausea and vomiting

*P < 0.05, **P < 0.001

## Discussions

This randomized clinical trial explored the effect of depth of anesthesia on postoperative pain in patients with obesity. According to the study's findings, maintaining a deeper depth of anesthesia in patients with obesity during surgery is beneficial to reduce acute postoperative pain, decrease the use of analgesia, and increase patient satisfaction.

In our study, the VAS score of the BIS 35 group was lower than that of the BIS 50 group. It has been observed that patients who received deep anesthesia tend to have lower postoperative pain scores, require less additional analgesic medication, and report higher levels of satisfaction on the first day after surgery. However, the pain levels measured using the VAS are similar among patients who received deep anesthesia and those who did not on the second and third day after the surgery. The results of the present study are comparable with those of Faiz et al., which shows that in the first twenty-four hours following laparoscopic cholecystectomy, deep anesthesia produced better pain outcomes than light anesthesia [12]. As a result, we may speculate that during surgery, deep anesthesia may have partially aborted noxious stimuli, affecting pain intensity and analgesic need.

We used BIS to monitor the depth of anesthesia, which has the best correlation with the blood concentration of propofol [5]. In the BIS 35 group, propofol consumption was considerably higher (*P* = 0.041). Some studies have shown that propofol plays an antinociceptive effect in the central nervous system through GABAA receptors and spinal delta opioid receptors, while exerting peripheral analgesic effects through its anti-inflammatory and antioxidant action [21–23]. This effect of suppressing noxious stimuli will gradually decrease with time, which explains the phenomenon that the deep anesthesia group experienced less pain following surgery and required fewer additional analgesic medications. On the second and third days, the VAS score was similar in both groups. The results of the present study are also comparable with a meta-analysis of randomized controlled trials, which reveals that deep anesthesia reduces early postoperative pain, but there was no difference in VAS pain score for persistent pain 3–12 months after surgery [24].

Some studies about the depth of anesthesia have shown that there is a difference in the MAP and vasoactive drug consumption [25]. Norepinephrine and nitroglycerin were used to regulate intraoperative blood pressure in this trial, and the doses of these drugs were comparable between two groups. Taking into account the difference in age of the participants, the main population in this study was young people, with better general conditions before surgery and a smaller range of hemodynamic fluctuations during surgery. Therefore, there was no difference in MAP and vasoactive drug use between the two groups.

In addition, some studies have shown that dexmedetomidine as an α_2_ adrenergic agonist can reduce the requirement for propofol and remifentanil, leading to hemodynamic stability during intravenous anesthesia and reduced postoperative pain [26, 27]. In this study, dexamethasone was used as one of the means to reduce postoperative nausea and vomiting. Moreover, studies have shown that dexamethasone has a role in reducing postoperative pain due to its powerful anti-inflammatory effect by inhibiting prostaglandin and aggregation of inflammatory corpuscle [28, 29]. Meanwhile, opioids and local anesthetic ropivacaine were used for intraoperative and postoperative analgesia but no significant difference in anesthetics (dexmedetomidine, opioids) or other drugs (dexamethasone, ropivacaine) was observed, which may not affect the postoperative VAS.

No differences in the grade of PONV at 24 h were observed in both groups, which conflicts with the results of previous experiments. According to the report of Sahni et al., the deep anesthesia group had fewer patients who experienced PONV at 0 and 8 h postoperatively [19]. However, in this trial, there was no difference in the incidence of PONV among different anesthesia depths. This may be due to the fact that the data of PONV were collected only 24 h after surgery and some differences were missed. Besides, the population in this study is at high risk of PONV, although preventive measures have been taken, the incidence was still high. The QoR-15 is a common score scale to measure the quality of recovery after surgery and anesthesia, including five dimensions: emotional state (4 items), physical comfort (5 items), psychological support (2 items), physical independence (2 items), and pain (2 items) [30]. In this study, no differences in the QoR-15 scores on 3 days postoperatively were observed in both groups, which is consistent with the result of Ning et al. [31]. This may be due to lower postoperative pain scores in the deep anesthesia group, but there was little difference in other dimensions. The proper dosage for a particular patient can decrease the duration in the operating room and PACU [32]. This study's findings showed that light anesthesia (BIS 50) could speed up anesthesia recovery and decrease the time needed for extubation, which was consistent with previous studies [31–33].

This study contains several limitations. First, in this study, only one scale was used to gauge the intensity of postoperative pain; in the future, other measures will be created to gauge the intensity of acute postoperative pain. Second, patients were only followed up at specific time points, and some positive events may be left out. Third, in this trial, the effect of anesthesia depth on postoperative pain was studied, while the preoperative pain of the patients was not statistically analyzed, which may lead to results bias. Last but not least, only acute postoperative pain was examined; further inquiry is necessary to evaluate chronic pain issues.

## Conclusions

For patients with obesity, maintaining a deeper depth of anesthesia during surgery is beneficial to reduce acute postoperative pain, cause less need for additional analgesic drugs, and improve patient satisfaction.

## Data Availability

The datasets used and/or analyzed during the study are available from the corresponding author on reasonable request.

## Declarations

### Clinical Trial Registration

Chinese Clinical Trial Registry (ChiCTR2300071313).
